# Use of postmenopausal hormone replacement therapy and risk of non-Hodgkin's lymphoma: a Danish Population-based Cohort Study

**DOI:** 10.1038/sj.bjc.6603123

**Published:** 2006-05-02

**Authors:** M Nørgaard, A H Poulsen, L Pedersen, H Gregersen, S Friis, M Ewertz, H E Johnsen, H T Sørensen

**Affiliations:** 1 Department of Clinical Epidemiology, Aarhus University Hospital, Sdr. Skovvej 15, Postbox 365, DK-9100 Aalborg, Denmark; 2 Institute of Cancer Epidemiology, Danish Cancer Society, DK-2100 Copenhagen, Denmark; 3 Department of Haematology, Aalborg Hospital, Aarhus University Hospital, DK-9000 Aalborg, Denmark; 4 Department of Oncology, Aalborg Hospital, Aarhus University Hospital, DK-9000 Aalborg, Denmark

**Keywords:** postmenopausal hormone replacement therapy, non-Hodgkin's lymphoma, population-based study, risk, cohort study

## Abstract

Use of postmenopausal hormone replacement therapy (HRT) has been hypothesised to be associated with a reduced risk of non-Hodgkin's lymphoma (NHL), but the epidemiologic evidence is conflicting. To examine the risk of NHL in HRT users aged 40 and older, we conducted a cohort study in the County of North Jutland, Denmark (population 0.5 million) using data from population-based health registries for the period 1989–2002. We computed age-standardised NHL incidence rates and used Cox regression analysis to compute the relative risk (RR) and corresponding 95% confidence intervals (CI) of NHL among HRT users compared with non-users, adjusting for age and calendar period. The number of prescriptions redeemed (1, 2–4, 5–9, 10–19, or 20 or more prescriptions) was used as a proxy for duration of HRT. We identified 40 NHL cases among HRT users during 179 838 person-years of follow-up and 310 NHL cases among non-users during 1 247 302 person-years of follow-up. The age-standardised incidence rates of NHL were 25.7 per 100 000 among HRT users and 24.2 per 100 000 among non-users, yielding an adjusted RR of 0.99 (95% CI: 0.71–1.39). Our data did not support an association between HRT use and risk of NHL.

The incidence of non-Hodgkin's lymphoma (NHL) has increased dramatically during the past 50 years in most Western countries (Hjalgrim *et al*, 1996; Zinzani, 2005). Established risk-factors, such as immunosuppresion, autoimmunity, and HIV infection account for only a small fraction of cases (Adami *et al*, 2002). Men are reported to have a higher incidence rates of NHL than women. In Denmark age-standardised incidence rates (based on the World Standard population) in the year 2000 were nine per 100 000 in men and seven per 100 000 in women (Danish Board of Health, 2004), and this difference may suggest a protective impact of female sex hormones. Supposedly, oestrogen and progesterone play a role in modulation of the immune system (Forsberg, 1984; Medina *et al*, 2000), but the mechanism is not clear.

Hormone replacement therapy (HRT) with oestrogens, with or without progestin, for menopausal symptoms has been associated with a decreased risk of Hodgkin's disease (Glaser *et al*, 2003). However, studies of HRT use and risk of NHL are few (Tavani *et al*, 1997; Nelson *et al*, 2001; Cerhan *et al*, 2002) and with varying results. We therefore conducted a cohort study using a population-based prescription database available in North Jutland, Denmark and the Danish Cancer Registry.

## MATERIALS AND METHODS

### Study population

We conducted this cohort study in North Jutland County, Denmark, which has nearly 500 000 inhabitants (approximately 9% of the Danish population). Using the files of the Civil Registration System, which includes all County residents, we identified 157 024 women aged 40 or older at any time in the period 1 January 1989 to 31 December 2002. The cohort comprised 149 132 women following exclusion of two groups: women with documented HRT use before the age of 40 (*n*=880) identified through linkage with the Pharmaco-Epidemiologic Prescription Database (see below); and women with a history of cancer (except nonmelanoma skin cancer) before study entry (pre-1989 or under the age of 40 years, *n*=7012), identified through linkage with the Danish Cancer Registry. The Danish Cancer Registry has recorded incident cases of cancer on a nationwide basis since 1943 and has been shown to have accurate and virtually complete ascertainment of cancer cases (Storm *et al*, 1997).

### Assessment of HRT use

The National Health Service (NHS) in Denmark provides tax-supported free health care for all inhabitants, guaranteeing free access to general practitioners and hospitals; the NHS refunds a varying proportion of costs of medication prescribed by physicians through a computerised accounting system. In North Jutland County, this accounting system also serves as the source of data for the Pharmaco-Epidemiologic Prescription Database, which was initiated in 1989 and covered all pharmacies in the county by 1991 (Gaist *et al*, 1997). This database includes the customer's civil registration number (a unique number assigned to all Danish residents that encodes gender and date of birth), the type and amount of drug prescribed according to the Anatomical Therapeutical Chemical (ATC) classification system, and the date the drug was dispensed. We used the prescription database to identify all prescriptions of HRT (ATC codes: G03A, G03C, G03D and G03F) during the study period. In Denmark, costs for oral contraceptives used for contraception are not reimbursed. Women who do receive reimbursement for the costs of oral contraceptives are using the medication for purposes other than contraception. This group of women was included in the study. Prescription of topical HRT was not included. We also retrieved information on prescriptions for sex hormones in addition to those used in the traditional HRT definition including androgens, antiandrogens, androgen antagonists and clomiphene (ATC codes: G03B, G03G, G03H and G03X).

### Information on NHL

Linkage to the Danish Cancer Registry (Storm *et al*, 1997) allowed us to identify cases of NHL diagnosed between 1 January 1989 and 31 December 2002 among women aged 40 and older. Follow-up to identify cases of NHL continued until the date of an NHL diagnosis, the date of a cancer diagnosis other than NHL (except nonmelanoma skin cancer), the date of a reimbursed prescription for non-HRT sex hormones, the date of death, the date of migration, or 31 December 2002, whichever came first.

### Data analysis

We computed age-standardised NHL incidence rates (standardised to the age distribution of the base population) as well as age-standardised NHL incidence rate ratios, defined as the NHL incidence rate for women using HRT divided by the NHL incidence rate for non-users. In addition, we used Cox's regression analysis to compute the hazard ratio as a measure of relative risk (RR) and associated 95% confidence intervals (CI) of NHL among HRT users compared with non-users, adjusting for age and calendar period. Hormone replacement therapy use was categorised into five groups based on number of prescriptions redeemed (1 prescription, 2–4 prescriptions, 5–9 prescriptions, 10–19 prescriptions, and 20 or more prescriptions) (Ewertz *et al*, 2005). We further stratified follow-up time according to years since the last HRT-prescription (less than 2, 2–5, and more than 5 years). Over time, the model allowed subjects to change between categories of covariates and exposure variables. Within each categorical level all variables were treated as time-independent.

## RESULTS

Among 23 708 HRT users we identified 40 NHL cases during a combined follow-up period of 179 838 person-years. Among 125 424 non-users, we identified 310 NHL cases during 1 247 302 person-years of follow-up. Age-standardised incidence rates of NHL were 25.7 per 100 000 person-years among HRT users and 24.2 per 100 000 person-years among non-users, yielding an age-standardised rate ratio of 1.06 (95% CI: 0.761.44). After adjusting for calendar period, the RR was 0.99 (95% CI: 0.71–1.39).

After stratifying by number of prescriptions, we found no substantial indication of decreased risk of NHL associated with HRT use. Age-standardised NHL rate ratios were 0.99 (95% CI: 0.36–2.16) among patients with one HRT prescription and 0.95 (95% CI: 0.47–1.69) among patients with more than 20 HRT prescriptions. Similarly, the adjusted RR estimates according to number of prescriptions were 0.91 (95% CI: 0.41–2.05) for one prescription, and 0.98 (95% CI: 0.53–1.80) for more than 20 prescriptions. In addition, we observed no trends in RR estimates with time since the last redeemed prescription (Table 1).

Among the 40 HRT exposed NHL cases four were classified as follicular lymphomas compared with 38 follicular lymphomas among the 310 unexposed NHL cases, yielding an adjusted RR on 0.56 (95% CI: 0.20–1.61). For large B-cell lymphoma the adjusted RR was 0.57 (95% CI: 0.27–1.18) based on 8 cases of large B-cell lymphomas among HRT users and 103 among non-users.

## DISCUSSION

This large population-based cohort study did not indicate any substantial association between HRT use and NHL risk. In contrast, an American case–control study involving 177 cases with intermediate- or high-grade B-cell NHL and 177 community controls found a decreased risk of NHL associated with HRT use (OR 0.64 (95% CI: 0.32–1.29)) (Nelson *et al*, 2001). These findings accorded with an Italian case-control study involving 145 NHL cases and 361 hospital controls, in which ‘ever use’ of HRT was associated with a decreased risk of NHL compared with ‘never use’ (OR 0.7 (95% CI: 0.3–1.4)) (Tavani *et al*, 1997). On the other hand, the Iowa Women's Health Study Cohort, which included 37 220 women and a total of 258 NHL cases, found that current HRT users were at increased risk of NHL (RR 1.4 (95% CI: 0.9–2.0). This association, however, was only seen in patients with follicular NHL (RR 3.3 (95% CI: 1.6–6.9)) and no associations were found between HRT use and risk of diffuse NHL (RR 1.1 (95% CI: 0.6–2.0)) or small lymphocytic NHL (RR 0.6 (95% CI: 0.1–2.7)) (Cerhan *et al*, 2002).

A number of reproductive factors have been studied in relation to NHL. In the Italian case–control study, the ORs of NHL by number of pregnancies, abortions, births, age at first birth, and time since last birth were all close to unity (Tavani *et al*, 1997). Adami *et al* (1997) found that associations between parity and NHL risk were weak – if they existed at all – and concluded that changes in reproductive patterns are unlikely to contribute to the increase in NHL. On the basis of our current findings, we can extend this conclusion to postmenopausal HRT use.

The main strengths of our study were its relatively large size and a well-defined geographical area, for which standardised population-based information was available on exposure as well as outcome variables. Exposure data included a full history of prescriptions for HRT during the study period. As HRT is only available by prescription in Denmark, we have probably captured virtually all women exposed to HRT in the study population during the follow-up period (Løkkegaard *et al*, 2004).

A potential study weakness is the lack of information on compliance in taking the prescribed medications. However, a comprehensive validation study in our setting, regarding self-reported HRT use among Danish nurses, found that more than 82% of those recorded in the database as ever-users reported having used HRT (Løkkegaard *et al*, 2004). Moreover, most of the patients in our study redeemed more than one prescription, and no substantial association was found between HRT use and risk of NHL even among patients who had redeemed more than 20 prescriptions.

The lacking information on other potentially confounding factors such as immunosuppresion, autoimmunity, and HIV infection was also a limitation (Levine and Hoover, 1992; Bhaskaran *et al*, 2004; Askling *et al*, 2005). Given the low prevalence of these diseases, however, their possible confounding effect is unlikely to have influenced the results substantially. We also lacked information about lifestyle and environmental exposures. In studies of HRT and primary prevention of cardiovascular disease, discordant results have been found between observational data and prevention trials (Humphrey *et al*, 2002). As a possible explanation for these discordant results selection bias and particularly healthy user bias has been suggested (Humphrey *et al*, 2002). It is possible that women with medical or psychiatric problems are less likely to receive HRT, and that women who are prescribed HRT are in better overall health, which could be associated with a decreased risk of lymphoma. We cannot rule out that this selection bias may have caused us to underestimate the effect of HRT. In North Jutland County, there is no strong association between the use of HRT and socioeconomic status (Olesen *et al*, 2005), and therefore we do not expect important confounding by socioeconomic status.

In conclusion, our data did not support any important association between HRT use and risk of NHL.

## Figures and Tables

**Table 1:** Risk of non-Hodgkin's lymphoma associated with hormone replacement therapy among women aged 40 years or older in North Jutland County, Denmark

	**Number of NHL cases**	**Person-years**	**Age-standardised incidence rate (/10^5^ person-years)**	**Standardised rate ratio (95% CI)**	**Adjusted relative risk^a^ (95% CI)**
Unexposed	310	1 247 302	24.2	Reference	Reference
HRT-exposed (1+ prescriptions)	40	179 838	25.7	1.06 (0.76–1.44)	0.99 (0.71–1.39)
					
*Recency*					
<2 years since latest prescription	25	119 914	22.9	0.95 (0.61–1.40)	0.98 (0.65–1.49)
2–5 years since latest prescription	6	33 127	22.5	0.93 (0.34–2.03)	0.78 (0.35–1.76)
>5 years since latest prescription	9	26 796	30.5	1.26 (0.58–2.39)	1.24 (0.63–2.42)
					
*Number of HRT prescriptions*					
1	6	29 292	24.0	0.99 (0.36–2.16)	0.91 (0.41–2.05)
2–4	5	37 507	16.0	0.66 (0.22–1.55)	0.63 (0.26–1.52)
5–9	8	32 437	34.7	1.43 (0.62–2.83)	1.16 (0.57–2.35)
10–19	10	35 463	33.2	1.37 (0.66–2.52)	1.31 (0.69–2.48)
20+	11	45 139	22.9	0.95 (0.47–1.69)	0.98 (0.53–1.80)

a Adjusted for age and calendar period.
